# Oral administration of potassium bromate induces neurobehavioral changes, alters cerebral neurotransmitters level and impairs brain tissue of swiss mice

**DOI:** 10.1186/s12993-016-0098-8

**Published:** 2016-05-12

**Authors:** Jamaan Ajarem, Naif G. Altoom, Ahmed A. Allam, Saleh N. Maodaa, Mostafa A. Abdel- Maksoud, Billy KC. Chow

**Affiliations:** Department of Zoology, College of Science, King Saud University, P.O. Box 2455, Riyadh, 11451 Saudi Arabia; Department of Zoology, Faculty of Science, Beni-suef University, Beni-Suef, Egypt; School of Biological Sciences, University of Hong Kong, Hong Kong, China

**Keywords:** Organ toxicity, Dopamine, Serotonin, Acetylcholine, Reduced glutathione

## Abstract

**Background:**

Potassium bromate (KBrO_3_) is widely used as a food additive and is a major water disinfection by-product. The present study reports the side effects of KBrO_3_ administration on the brain functions and behaviour of albino mice.

**Methods:**

Animals were divided into three groups: control, low dose KBrO_3_ (100 mg/kg/day) and high dose KBrO_3_ (200 mg/kg/day) groups.

**Results:**

Administration of KBrO_3_ led to a significant change in the body weight in the animals of the high dose group in the first, second and the last weeks while water consumption was not significantly changed. Neurobehavioral changes and a reduced Neurotransmitters levels were observed in both KBrO_3_ groups of mice. Also, the brain level of reduced glutathione (GSH) in KBrO_3_ receiving animals was decreased. Histological studies favoured these biochemical results showing extensive damage in the histological sections of brain of KBrO_3_-treated animals.

**Conclusions:**

These results show that KBrO_3_ has serious damaging effects on the central nervous system and therefore, its use should be avoided.

## Background

Potassium bromate (KBrO_3_) is widely used as a flour improver that acts as a maturing agent [1]. During the last 90 years, it has been used as a food additive [2]. It acts principally in the late dough stage giving strength and elasticity to the dough during the baking process in addition to promoting the rise of bread. KBrO_3_ is also used in cheese production, beer making and is commonly added to fish paste products [3]. Also, it is used in pharmaceutical and cosmetic industries and is a constituent of cold wave hair solutions [2]. Moreover, KBrO_3_ can appear as a byproduct in an ozonization of water containing bromide. As a result of KBrO_3_ biotransformation, free radicals’ generation can cause oxidative damage to essential cellular macro molecules, leading to marked nephrotoxicity and cancer in experimental animals [4]. Indeed, many previous reports have documented that KBrO_3_ can induce multiple organ toxicity in humans and experimental animals [5–7]. KBrO_3_ is highly irritating and injurious to tissues especially those of the central nervous system (CNS) and kidneys [8]. Many cases of accidental poisoning in children resulting from ingestion of bromate solution and sugar contaminated with bromate were reported as the source of mild poisoning in New Zealand [9]. Consequently, KBrO_3_ has been prohibited in several countries like United Kingdom, Nigeria and Canada [2]. Toxicological studies have convincingly shown that KBrO_3_ affects the neurobehavioral (motor equilibrium performance and spontaneous locomotor activity) status of guinea pigs [10]. KBrO_3_ induced detrimental effects on auditory brainstem response of guinea pigs whereas it caused Otto-neurotoxicity mainly through the peripheral auditory nerve [11]. Behavioral changes are usually associated with a disturbance in neurotransmitters [12]. Acetylcholine, dopamine and serotonin are common neurotransmitters that can directly or indirectly influence neurons, thereby affecting behavior [13]. Behavioral changes are also associated with oxidative stress [14]. It is known that KBrO_3_ induces oxidative stress in tissues [15–18] that could be the basis of bromate-induced behavioral changes. Moreover, KBrO_3_ induces hemorrhage, neuronal degeneration and vacuolation of the brain tissue sections [19]. The present study attempts to assess the effect of oral administration of KBrO_3_ on the behavioral changes, neurotransmitters, antioxidant status and brain histomorphology of white albino mice using two different doses of KBrO_3_ to compare their effects.

## Methods

### Animals

Thirty (30) adult male albino mice (*Mice musculus*) with an average weight of 30.2 ± 4.24 g were obtained from animal house- College of pharmacy- King Saud University and maintained and monitored in a specific pathogen-free environment. All animal procedures were performed in accordance with the standards set out in the Guidelines for the Care and Use of Experimental Animals issued by the Committee for the Purpose of Control and Supervision of Experiments on Animals (CPCSEA). The study protocol was approved by the Animal Ethics Committee at King Saud University. All animals were allowed to acclimatize in plastic cages inside a well-ventilated room for one week prior to the experiment. The animals were maintained under standard laboratory conditions (temperature of 23 °C, relative humidity of 60–70 % and a 12-hour light/dark cycle), fed a diet of standard commercial pellets and given water ad libitum.

### Potassium bromate preparation and dosing schedule

Potassium bromate salt, a product of British drug home limited, Poole England was supplied in its white crystalline form by ASILA chemicals (Saudi Arabia). It was then dissolved in water to prepare the 100 mg/kg dose and the 200 mg/kg dose. Animals were divided into 3 groups as follows: Group (I) control group (was given distilled water); Group (II) Low dose KBrO_3_ group (was given 100 mg/kg); Group (III) High dose KBrO_3_ group (was given 200 mg/kg). KBro_3_ was orally administered daily through oral intubation at the two doses of 100 and 200 mg/kg/day for 42 days. The doses used in the current study were adjusted according to the LD50 calculations carried out by Kurokawa et al. [24].

### Monitoring of water consumption and body weight changes

Daily water consumption was monitored for all animals in the three groups. The animals were weighed prior to the commencement of administration and in subsequent weeks during the experiment period. At the end of administration, the mice were sacrificed by cervical dislocation.

### Behavioral studies

Ten animals from each group were used in the current study. For testing, the animals were brought in a room (25 °C) of dim red light reserved for that purpose. All tests were conducted blindly by the same experimenter [20]. Except for Morris maze experiment, all experiments were carried out during the 3rd and the 6th weeks.

#### T-maze conducting assay

All animal’s groups were deprived from food all night before this examination. The elevated T-maze consists of three closed arms to be T like structure. The main arm (100 × 10 × 20 cm) and the two lateral arms (40 × 10 × 20 cm) at an elevation of 20 cm above the floor. At the end of the right lateral arm, the rodent food was placed. Hungry animals were placed in the terminal end of the main arm of the elevated T-maze facing the passage to the two lateral arms, and left to explore the maze for one min then the animal removed from the maze and kept in its cage for 2 hrs and replaced in the same position in the main arm and the behavior analyzed for 5 min. Both of the time spent exploring the arms to reach food, and the time spent in the food arm in seconds, were determined. The frequency and time of entering the food lateral arm was considered to be memory reflector.

#### Grip-strength meter assay

The Ugo Basile 47,200-Grip-Strength Meter (COMERIO-Varese, Italy) is suitable for mice and can automatically measures grip-strength (i.e. peak force and time resistance) of forelimbs in mice. The aim was to assess forelimbs muscle strength. Each animal was tested three times and the peak force of each mouse was recorded. The mean of three values of each mouse was recorded.

#### Rota-rod assay

The Ugo Basile Rota-rod instrument (COMERIO-Varese, Italy) has been used in this test. The mouse is placed on a horizontally oriented position and mechanically rotating at 15 rpm rod. The rod is suspended above a cage floor, which is high enough for avoidance of fall. Mice naturally try to stay on the rotating rod, or Rota-rods, and avoid falling to the ground. The length of time that a given animal stays on this rotating rod is considered as a measure of their balance, coordination, and motor-activity.

### Sample collection

For histological studies, brains were removed and cut into small pieces in sterile saline solution, fixed in 10 % neutral buffered formalin and embedded in paraffin. For biochemical investigations, samples were prepared by weighing 200 mg of longitudinal brain sections into a dry and clean Teflon digestion beaker, to which, 6 ml of HNO_3_, 2 ml HCl and 2 ml HF were added. Samples were digested on the hot plate at 120–150 °C for 40 min. The resulting digest was filtered through whatman filtered paper no42. The filtered digest was transferred to a 50 ml plastic volumetric flask and completed to the mark using deionized water.

### Histological studies

For the histological slides preparation, left loop of cerebellum, cerebral cortex and medulla oblongata of three sacrificed animals were fixed in 20 % formalin saline for 24 h. To remove the excess of the fixative, the tissues were washed and then dehydrated in ascending grades (70, 80, 90 and 95 %) of ethanol for 45 min each, then in two changes of absolute ethanol for 30 min each. This was followed by two changes of xylene for 30 min each. The tissues were then impregnated and embedded in paraplast plus. Sections (4–5 µm) were prepared with a microtome, de-waxed, hydrated and stained in Mayer’s haemalum solution for 3 min. The sections were stained in Eosin for one min, washed in tap water and dehydrated in ethanol as described above.

### Neurochemical studies

#### Dopamine and serotonin determination

The level of dopamine and serotonin was estimated in the brain using the modified method of Patrick et al. [21]. A 10 % homogenate of the brain has been re-centrifuged at 17,000 rpm at 4 °C for 5 min. The supernatants were filtered using 0.45 μm pore filters and analyzed by high performance liquid chromatography. The mobile phase consisted of 32 mM citric acid monohydrate, 12.5 mM disodium hydrogen orthophosphate, 7 % methanol, 1 mM octane sulfonic acid and 0.05 mM EDTA. The mobile phase was filtered through 0.22 μm filter and degassed under vacuum before use. Bondpak C18 column was used at a flow rate of 1.2 ml/min and the injection volume of the sample was 20 μl. The levels of dopamine and serotonin were calculated using a calibration curve and results were expressed as ng/mg tissue weight.

#### Determination of acetylcholine

The level of acetylcholine was estimated in the brain using a method that has been described previously [22]. In brief, dialysate samples were injected into the liquid chromatography/electrochemistry system assisted by a chromatography manager (Millennium; Waters, Milford, MA), and analyzed for acetylcholine. Acetylcholine was separated on a coiled cation exchanger acetylcholine column (analytical column) (Sepstik 530 × 1.0 mm I.D., packed with polymetric strong exchanger, 10 μm in diameter; BAS, West Lafayette, IN), followed by the post-immobilized enzyme reactor which consisted of choline oxidase/acetylcholine esterase. Acetylcholine was hydrolyzed by acetylcholine esterase to form acetate and choline in the post-immobilized enzyme reactor, and then choline was oxidized by choline oxidase to produce betaine and hydrogen peroxide (H_2_O_2_). H_2_O_2_ is detected via oxidation of horseradish peroxidase, which in turn entrapped in the redox polymer coated on the surface of the glassy carbon electrode (MF-9080; BAS), set at +100 mV (LC-4C; BAS) versus Ag/AgCl reference electrode. This reduction was analyzed with the detector (LC-4C; BAS) as a signal indicating acetylcholine on the chromatogram.

#### Reduced Glutathione (GSH) assay

Reduced glutathione content was determined according to the method of Beutler et al. [23] with some modification. Briefly, 0.20 ml of tissue supernatant was mixed with 1.5 ml of the precipitating solution which contains 1.67 % glacial metaphosphoric acid, 0.20 % Na-EDTA and 30 % NaCl. The mixture was allowed to stand for 5 min at room temperature and centrifuged at 1000 rpm for 5 min. One ml clear supernatant was mixed with 4 ml 0.30 M Na_2_HPO_4_ and 0.50 ml DTNB reagent (40 mg 5, 5′dithiobis-(2-nitrobenzoic acid dissolved in 1 % sodium citrate). The blank was prepared similarly whereas 0.20 ml water was used instead of the brain supernatant. The absorbance of the color was measure at 412 nm in a spectrophotometer.

### Statistical analysis

Prior to further statistical analysis, the data were tested for normality using the Anderson–Darling test, as well as for homogeneity variances. The data was normally distributed and is expressed as the mean ± standard error of the mean (SEM). Significant differences among the groups were analysed by one- or two-way ANOVA followed by Tukey’s post-test using SPSS software, version 17. Differences were considered statistically significant at P < 0.05.

## Results

### Effect of KBrO_3_ on the body weight and water consumption of the treated mice

**Fig. 1:**
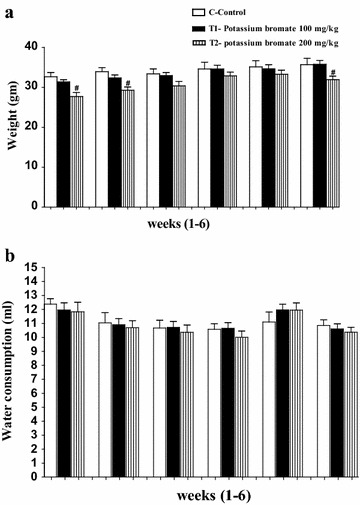
Effect of KBrO_3_ on the mean of body weight (gm) (**a**) and water consumption (ml) (**b**) in treated mice during six successive weeks using two different doses of KBrO_3_. The data are the mean ± SEM for 10 mice per group for the control group (*open white bars*), low dose KBrO_3_ treated group (*closed black bars*), and high dose KBrO_3_ treated group (*hatched bars*). *P < 0.05 for low dose KBrO_3_ treated group vs. control group; ^#^P < 0.05 for high dose KBrO_3_ treated group vs. control group; ^+^P<0.05 for high dose KBrO_3_ treated group vs. low dose KBrO_3_ treated group

Figure 1a illustrates that during the first, the second and the last weeks of KBrO_3_ treatment, the high dose of KBrO_3_ (200 mg/kg) was accompanied with a decrease in body weight in comparison to both the control group and the low dose treated group. On the other hand, the low dose of KBrO_3_ (100 mg/kg) effect on body weight decrease, was not significant. Water consumption was investigated to study its correlation with KBrO_3_ dose. As illustrated in Fig. 1b, the means of water consumption were similar between all KBrO_3_ exposure and control groups throughout the study.

### KBrO_3_ treatment can cause a disturbance in the behavior of the treated mice

**Fig. 2:**
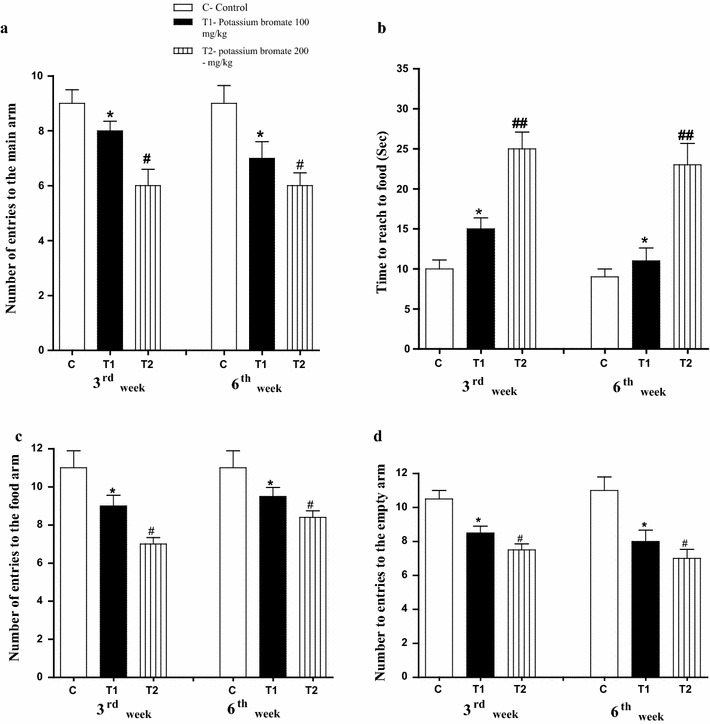
Effect of KBrO_3_ on the animal’s behavior in T-maze. **a** The number of entrances of main arm. **b** The time consumed to reach to the food. **c** The number of entrances to the food arm. **d** The number of entrances to the empty arm. Data are expressed as mean ± SEM for 10 mice per group

**Fig. 3:**
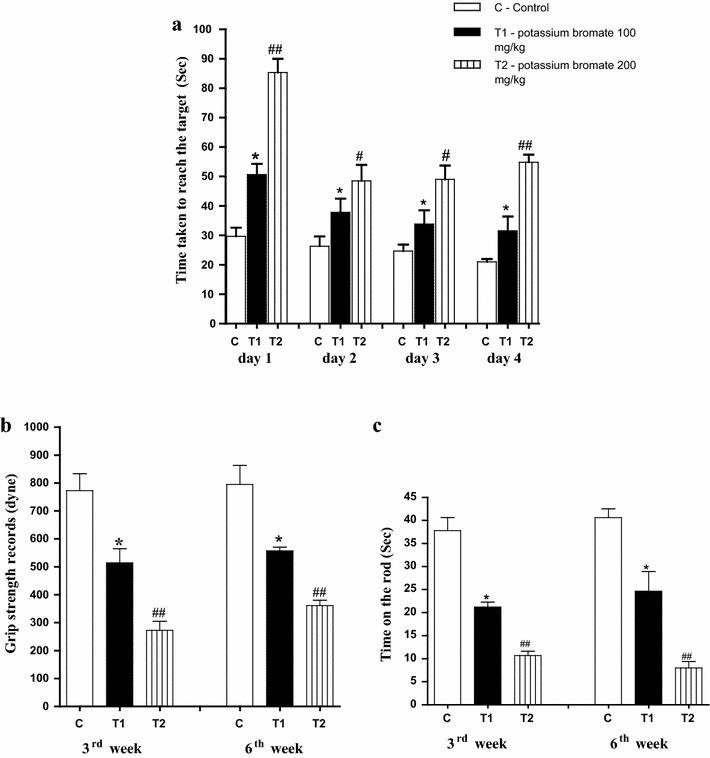
Effect of KBrO_3_ on **a** The animal’s behavior in water maze (Morris maze), **b** the fore limb grip strength records and **c** rota rod records for the animals of each group. Data are expressed as mean ± SEM for 10 mice per group

When investigating the behavior of animals in the T-maze, bad memory and low smell ability of the KBrO_3_ treated groups during both the 3rd week and the 6th week in comparison to the control group was recorded. This was represented in the reduction of the number of entrances to the main arm (Fig. 2a), the increase in the time consumed to reach to the food in the food arm (Fig. 2b), the decrease in both of the number of entrances to the food arm (Fig. 2c) and the number of entrances to the empty arm (Fig. 2d). Additionally, the Moris—maze examination confirmed the observed bad memory in T-maze. Learning ability was also limited in both of the KBrO_3_ groups whereas they consumed much time to reach to the target (Fig. 3a) along four successive days. Still, the harmful effect of the high dose of KBrO_3_ is much more significant than that of the low dose one.

The fore-limb muscles of the animals in the group of the KBrO_3_ low dose recorded lower beaks in comparison to the control group in the grip strength examination scores. Moreover, the recorded beaks of the high dose KBrO_3_ group appeared significantly lower than the control group during both the 3rd and the 6th weeks (Fig. 3b).

In rotator test, both of the KBrO_3_ groups have exhibited a short time on the rod during both the 3rd and the 6th weeks in comparison to the animals of the control group. The staying times of the animals of the low dose KBrO_3_ group on the rod were less than the time of the control group. For the high dose KBrO_3_ group, the staying times of the animals on the rod were lower than that of the low dose group (Fig. 3c).

### KBrO_3_ treatment is associated with depletion of neurotransmitters in the brain of the treated mice

**Fig. 4:**
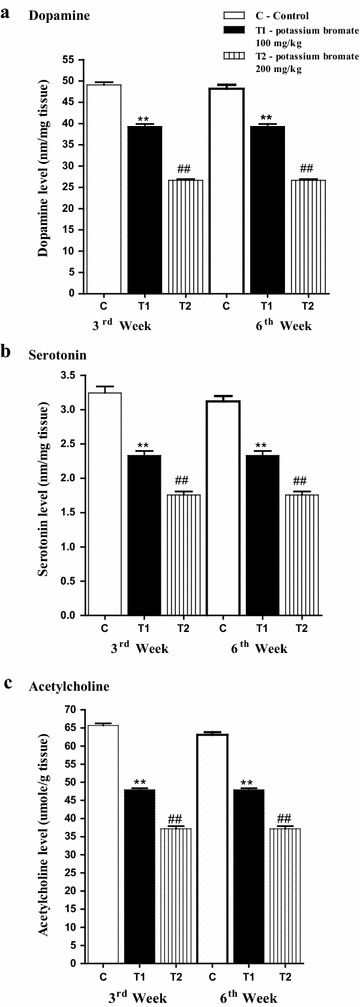
Effect of KBrO_3_ treatments on the level of neurotransmitters in the brain of treated mice. **a** Mean of Dopamine (nm/mg tissue) in brain tissue of treated mice during third and sixth weeks, **b** mean of Serotonin (nm/mg tissue) in brain tissue of treated mice during third and sixth weeks, **c** mean of Acetylcholine (umole/g tissue) in brain tissue of treated mice during third and sixth weeks. Data are expressed as mean ± SEM for 10 mice per group

Dopamine is an important neurotransmitter that plays a number of important roles in the brain. Consequently, investigating the brain level of this molecule after KBrO_3_ treatment is of special relevance. In comparison to the control group (49.089 ± 0.634), a significant (*P < *0.05*) depletion of dopamine concentration (39.338 ± 0.533) has been detected in the low dose KBrO_3_ group. Also, a highly significant (*#P* < *0.05*) reduction (26.672 ± 0.672) in the dopamine level in the high dose KBrO_3_ group in comparison to the control group was recorded (Fig. 4a). Another important monoamine neurotransmitter is serotonin. Here, a significant (*P < *0.05*) reduction (2.331 ± 0.0664) in brain-serotonin concentration in the low dose KBrO_3_ group in comparison to the control group (3.243 ± 0.0943) was detected. Additionally, a highly significant (^*#*^*P* < *0.05*) decrease (1.756 ± 0.0525) in brain-serotonin concentration in the high dose KBrO_3_ group in comparison to the control group was detected (Fig. 4b). Another organic molecule that acts as a neurotransmitter is acetylcholine. Indeed, acetylcholine concentrations showed similar results to that of both dopamine and serotonin. In comparison to the control group (65.678 ± 0.627), acetylcholine concentration in the brain of the low dose KBrO_3_ group was significantly (*P < *0.05*) reduced (47.936 ± 0.459). Again, the brain level of acetylcholine in the high dose KBrO_3_ group was significantly (^*#*^*P* < *0.05*) lower (37.16 ± 0.728) than that of both the control group and the low dose KBrO_3_ group (Fig. 4c). This harmful effect of KBrO_3_ was dominant during both the 3rd and the 6th weeks.

### Decreased brain level of reduced glutathione (GSH) after KBrO_3_ treatment in animals

**Fig. 5:**
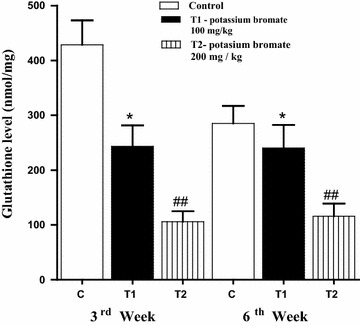
Effect of KBrO_3_ treatments on the level of GSH in the brain of treated mice. The brain level of GSH was determined during the third and the sixth weeks. Data are expressed as mean ± SEM for 10 mice per group. *P < 0.05 for low dose KBrO_3_ treated group vs. control group; ^#^P < 0.05 for high dose KBrO_3_ treated group vs. control group; ^+^P<0.05 for high dose KBrO_3_ treated group vs. low dose KBrO_3_ treated group

Reduced glutathione (GSH) is an important antioxidant that plays a crucial role in nearly all living organisms. KBrO_3_ treatment had a negative effect on the brain level of this important molecule. In the low dose KBrO_3_ group, the brain level of GSH was significantly (*P < *0.05*) reduced (250.31 ± 39.61) in comparison to the control group (420.63 ± 59.61). Also, a significant (^*#*^*P* < *0.05*) reduction in the level of this crucial molecule was detected in the high dose KBrO_3_ group (110.76 ± 15.87) in comparison to either the control group or the low dose KBrO_3_ group (Fig. 5). Like neurotransmitters, the reducing effect of KBrO_3_ on the glutathione level, was dominant during both the 3rd and the 6th weeks.

### KBrO_3_ treatment has induced brain histopathological changes in mice

**Fig. 6:**
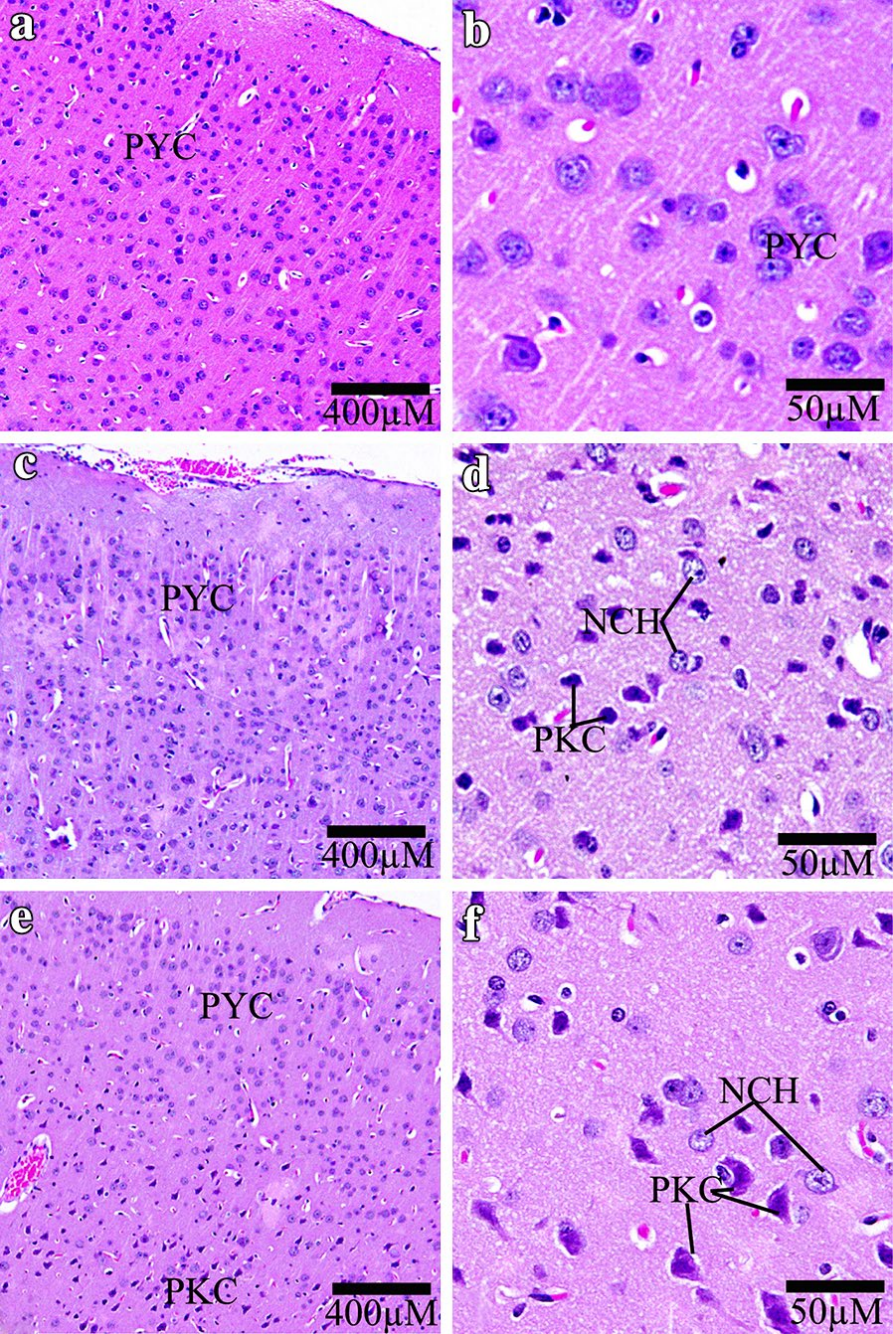
Effect of KBrO_3_ treatments on the Cerebral cortex of treated mice. Sagittal sections of the cerebral cortex depicting the pyramidal cell distribution (PYC), neurocytechromatolysis (NCH) and pyknosis (PKC) in the following groups: **a**, **b** control group, **c,** **d** potassium bromate 100 mg/kg group, **e**, **f** potassium bromate 200 mg/kg group. *Scale bar* 400 μm in **a**, **c**, **e** and 50 μm in **b**, **d**, **f**

In the control group, the normal pyramidal neurons exhibited their general characteristic shape with rounded, large and centrally located nuclei (Fig. 6). The normal cells of the cerebral cortex had a spherical perikaryon whose nuclei were large. Also the neurons were arranged in a regular pattern (Fig. 6a, b). In both of KBrO_3_ treated groups, pyknosis and chromatolysis have been observed in the pyramidal neurons (Fig. 6c, d, e, f).

**Fig. 7:**
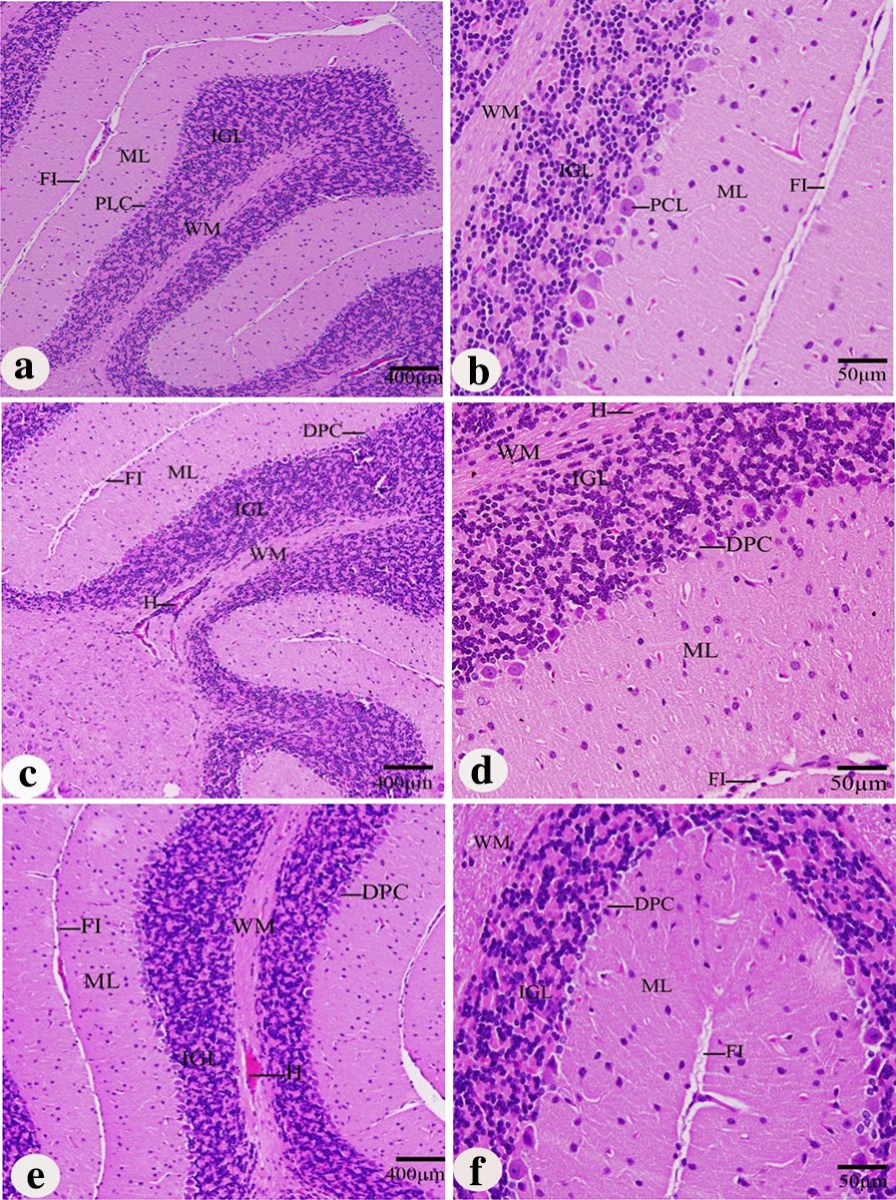
Effect of KBrO_3_ treatments on the Cerebellum of treated mice. Sagittal sections in the cerebellum cortex showing the degenerated Purkinje cell (DPC), fissure (FI), hemorrhage (H), internal granular layer (IGL), molecular layer (ML), Purkinje cell layer (PCL) and white matter (WM). **a**, **b** control group, **c**, **d** potassium bromate 100 mg/kg group, **e**, **f** potassium bromate200 mg/kg group. *Scale bar* 400 μm in **a**, **c**, **e** and 50 μm in **b**, **d**, **f**

**Fig. 8:**
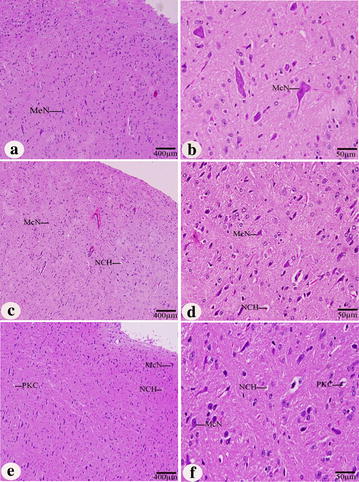
Effect of KBrO_3_ treatments on the medulla oblongata of treated mice. Sagittal sections in the medulla oblongata showing the medulla neurons (MeN), neurocytechromatolysis (NCH) and pyknosis (PKC). **a**, **b** control group, **c**, **d** potassium bromate 100 mg/kg group, **e**, **f** potassium bromate 200 mg/kg group. *Scale bar* 400 μm in **a**, **c**, **e** and 50 μm in **b**, **d**, **f**

In the cerebellum, the neuronal density in the molecular layer of control group was the highest compared to KBrO_3_ treated groups. The normal Purkinje cells were arranged in a single row of large neurons with pear-shaped perikaryon and large nucleus. The lateral processes disappeared and the apical processes formed the permanent dendritic tree (Fig. 7a, b). On the other hand, in both of KBrO_3_ treated groups, some degenerated Purkinje cells were detected and some were more spindle-shaped and small (Fig. 7c, d, e, f). In control group, the normal neurons in the medulla appeared large in size, polygonal, varied in shape and had round nuclei (Fig. 8a, b). In both of KBrO_3_ treated groups, most of medulla neurons appeared small and pyknotic (Fig. 8c, d, e, f). Also, degenerated medullary neurons were observed (Fig. 8 d, f).

## Discussion

Potassium bromate (KBrO_3_) is widely used as improving additive for bread making [7] and marketed as a neutralizer in home permanent cold wave hair kits. Several cases of accidental poisoning in children resulting from the ingestion of KBrO_3_ solution, were reported [9]. Due to its hazardous effects, it has been forbidden in various countries [2]. Toxicity studies in animals are commonly used to assess potential health risk in humans caused by intrinsic adverse effects of chemical compounds [24]. These adverse effects may manifest significant alterations in the levels of bio molecules, normal functioning and histomorphology of the organs [3]. The current study was designed to investigate some of the behavioral and biochemical changes induced by KBrO_3_ intake in albino mice. We have observed that oral intubation of KBrO_3_ at the dose of 200 mg/dl was accompanied with an obvious decrease in the body weight of the animals while the lower dose cannot do this effect. This is in agreement with the results obtained by Kurokawa et al. [25] who have reported a dose-dependent inhibition of body weight increase in both male and female F344 rats after oral administration of KBrO_3_. Also, similar results were obtained with guinea pigs [11]. Water consumption was not affected by the oral administration of KBrO_3_ with either the 100 or 200 mg/dl doses. These results agree with that of Dodd et al. [26] who have reported that only the 400 mg/L dose can result in a significant increase in water consumption and other lower doses cannot. Many environmental contaminants were reported to be associated with behavioral changes and this was elucidated in many studies before [27–31]. The abnormal pattern in open-field, social, learning and emotional behaviors were documented in Wister rats after receiving Sodium nitrite in the drinking water [32]. KBrO_3_-mediated behavioral changes seen in the current study may be attributed partially to the harmful effect of KBrO_3_ on the brain level of neurotransmitters. It was reported that abnormalities in the regulation of neurotransmitter release and/or abnormal levels of extracellular neurotransmitter concentrations are considered as core components of hypotheses on the neuronal foundations of behavioral and cognitive disorders and the symptoms of neuropsychiatric and neurodegenerative disorders [13]. GSH is the most abundant antioxidant molecule that is critical for protecting the brain from oxidative stress, acting as a free radical scavenger and inhibitor of lipid peroxidation. In the current study, the decrease in the brain level of the antioxidant molecule, GSH, could be considered as an important reason for the observed behavioral changes. Previous reports have documented a direct relationship between behavioral changes and oxidative stress [33, 34]. Oxidative stress can mediate neurodegeneration in hippocampus and behavioral changes of adult rats [35]. Concomitantly, KBrO_3_ induced pathological changes on the histological level in the brain tissue of the treated rats which may be considered as another important causative factor for the negative behavioral changes. Previous studies have reported hemorrhage, neuronal degeneration and vacuolation of the brain tissue sections of rats after KBrO_3_ treatment [19]. Taken together, our data illustrate that oral administration of KBrO_3_ has a direct effect on the behavioral level, neurotransmitters content, antioxidant status and brain histomorphology of white albino rat and that using two different doses of KBrO_3_ has different outcomes.

## Conclusions

Potassium bromate has deleterious effects on the central nervous system of mice. It can disturb the neurotransmitters levels, antioxidant defence molecules and induce histopathological changes in cerebral tissue. Therefore, its use in human used- products should be stopped.

## Abbreviations

EDTA: ethylene diamine tetra acetic Acid
ICP-MS: inductively coupled plasma mass spectrometer
GSH: reduced glutathione
KBrO_3_: potassium bromate
CNS: central nervous system
H_2_O_2_: hydrogen peroxide
